# Particulate matter air pollution and respiratory symptoms in individuals having either asthma or chronic obstructive pulmonary disease: a European multicentre panel study

**DOI:** 10.1186/1476-069X-11-75

**Published:** 2012-10-05

**Authors:** Anna Karakatsani, Antonis Analitis, Dimitra Perifanou, Jon G Ayres, Roy M Harrison, Anastasia Kotronarou, Ilias G Kavouras, Juha Pekkanen, Kaarle Hämeri, Gerard PA Kos, Jeroen J de Hartog, Gerard Hoek, Klea Katsouyanni

**Affiliations:** 1 2nd Department of Respiratory Medicine, “ATTIKON” University Hospital, Medical School, National and Kapodistrian University of Athens, 124 62, Haidari, Greece; 2 Department of Hygiene, Epidemiology and Medical Statistics, Medical School, National and Kapodistrian University of Athens, 115 27, Goudi, Athens, Greece; 3 Institute of Occupational and Environmental Medicine, University of Birmingham, Birmingham, United Kingdom; 4 Division of Environmental Health and Risk Management, School of Geography, Earth and Environmental Sciences, University of Birmingham, Edgbaston, Birmingham, B152TT, United Kingdom; 5 Department of Environmental Sciences / Center of Excellence in Environmental Studies, King Abdulaziz University, PO Box 80203, Jeddah, 21589, Saudi Arabia; 6 National Observatory of Athens, Institute for Environmental Research and Sustainable Development, Athens, Greece; 7 Department of Environmental Health, National Institute for Health and Welfare, Kuopio, Finland; 8 Public Health and Clinical Nutrition, University of Eastern Finland, Kuopio, Finland; 9 Department of Physics, University of Helsinki, Helsinki, Finland; 10 Energy research Center of the Netherlands, Environment and Energy Engineering, Environmental Assessment, Petten, The Netherlands; 11 University of Utrecht, Institute for Risk Assessment Sciences, Utrecht, The Netherlands

**Keywords:** Air pollution, Asthma, Chronic obstructive pulmonary disease, Coarse particles, Particle number concentration, Respiratory health

## Abstract

**Background:**

Particulate matter air pollution has been associated with adverse health effects. The fraction of ambient particles that are mainly responsible for the observed health effects is still a matter of controversy. Better characterization of the health relevant particle fraction will have major implications for air quality policy since it will determine which sources should be controlled.

The RUPIOH study, an EU-funded multicentre study, was designed to examine the distribution of various ambient particle metrics in four European cities (Amsterdam, Athens, Birmingham, Helsinki) and assess their health effects in participants with asthma or COPD, based on a detailed exposure assessment. In this paper the association of central site measurements with respiratory symptoms and restriction of activities is examined.

**Methods:**

At each centre a panel of participants with either asthma or COPD recorded respiratory symptoms and restriction of activities in a diary for six months. Exposure assessment included simultaneous measurements of coarse, fine and ultrafine particles at a central site. Data on gaseous pollutants were also collected. The associations of the 24-hour average concentrations of air pollution indices with the health outcomes were assessed in a hierarchical modelling approach. A city specific analysis controlling for potential confounders was followed by a meta-analysis to provide overall effect estimates.

**Results:**

A 10 μg/m^3^ increase in previous day coarse particles concentrations was positively associated with most symptoms (an increase of 0.6 to 0.7% in average) and limitation in walking (OR= 1.076, 95% CI: 1.026-1.128). Same day, previous day and previous two days ozone concentrations were positively associated with cough (OR= 1.061, 95% CI: 1.013-1.111; OR= 1.049, 95% CI: 1.016-1.083 and OR= 1.059, 95% CI: 1.027-1.091, respectively). No consistent associations were observed between fine particle concentrations, nitrogen dioxide and respiratory health effects. As for particle number concentrations negative association (mostly non-significant at the nominal level) was observed with most symptoms whilst the positive association with limitation of activities did not reach the nominal level of significance.

**Conclusions:**

The observed associations with coarse particles are in agreement with the findings of toxicological studies. Together they suggest it is prudent to regulate also coarse particles in addition to fine particles.

## Background

Over the last decades numerous epidemiological studies have clearly shown that urban air pollution can produce a variety of adverse health effects [1,2]. Ambient particulate matter (PM) either characterized as the mass concentration of particles less than 10 μm (PM_10_) or less than 2.5 μm (PM_2.5_) are considered to be the major culprit. Therefore, current air quality standards or guidelines refer to PM_10_ and/or PM_2.5_[3,4]. However, in reality ambient PM is a mixture of coarse (2.5-10 μm), PM_2.5_ (named also fine particles) and ultrafine (<0.1 μm) particles generated from different processes, having variable chemical composition and atmospheric behavior. It should also be noted that although the ultrafine fraction accounts for less than 1% of the mass of particulate matter, it represents the greatest proportion in terms of number of particles (typically >80%) [5-7]. Furthermore, the mechanism and the fraction of PM that are mainly responsible for the observed health effects is a matter of controversy [1]. In 1995 Seaton hypothesized that the number of ultrafine particles may be a more health relevant property than the usually measured mass of inhaled PM_10_ and PM_2.5_[8]. This is because of the greater surface area available to react with epithelial and inflammatory cells in the lung and because of the capacity of ultrafine particles to penetrate deeper in the lung parenchyma, potentially reaching the circulation and exerting adverse biological effects by releasing toxic free radicals [8-11]. In meantime other studies were published, however, the role of ultrafine particles is still under discussion [9,12-14].

The only systematic review of studies that have analysed fine and coarse PM jointly demonstrates that the health effects of coarse particles are significant and should not be overlooked [15]. Thus, special consideration should be given to each fraction of the particles and their effects on health. Better characterization of the health relevant particle fraction will have major implications for air quality policy since it will determine which sources should be controlled.

The RUPIOH (Relationship between Ultrafine and fine Particulate matter in Indoor and Outdoor air and respiratory Health) is an EU-funded multicentre study designed to examine the distribution of various particle metrics both indoors and outdoors in four European cities and assess their health effects in individuals with asthma or chronic obstructive pulmonary disease (COPD), based on a detailed exposure assessment. The study consisted of two parts: i) the diary study in which participants were asked to complete a daily diary for six months while exposure was assessed based on a central site measurements and ii) the intensive week measurements during which, for each subject, more intensive health and exposure measurements were conducted. In this paper, we report the association of ambient PM_10_, PM_2.5_, coarse particle mass (PM_10-2.5_) and particle number concentrations (PNC), measured at the central site, with respiratory symptoms and limitation in activities due to breathing problems in participants having either asthma or COPD who have been followed for six months. Associations of the health outcomes with gaseous air pollutants were also examined based on data collected from existing national monitoring networks in each country. The relationships between central site outdoor, residential outdoor and indoor concentrations, as well as the association between outdoor and indoor exposure to fine and ultrafine particles and lung function in the same participants but based on the intensive week measurements have been published before [16-20].

## Methods

### Study design

In the context of RUPIOH, a multicentre study was conducted from October 2002 to March 2004 in four European metropolitan areas, namely, Amsterdam (The Netherlands), Athens (Greece), Birmingham (United Kingdom) and Helsinki (Finland). During the whole study period a central site in each city was used to monitor particle mass and PNC on a daily basis. At various locations covering the entire metropolitan area, homes of participants with either asthma or COPD were selected. The criteria for the central site and homes selection have been described in detail in a previous publication [17]. Respiratory health status of each participant was monitored for six months by a daily symptom diary. We used a staged entry of the participants (based on the real date the participants started to fill out the diaries) in order to increase the period of data collection and thus, decrease the likelihood for uncontrolled factors or unexpected events to influence the associations between air pollution and health [21]. In all centres, participants were recruited between October 2002 and March 2004.

### Study population

Inclusion criteria and recruitment procedures have been described in detail before [19]. Briefly, in each city the recruitment criteria for participants were age 35 or more, a doctor diagnosis of either asthma (as defined by Global Initiative for Asthma) or COPD (as defined by Global Initiative for Chronic Obstructive Lung Disease) and having had experienced respiratory symptoms in the past 12 months [22,23]. Especially, in the Netherlands some patients who had not received a definite diagnosis of asthma or COPD were classified as chronic non-specific lung disease (CNSLD) as a relic of tradition (term previously used to indicate either asthma or COPD) [24]. Severe patients defined as those using relief bronchodilating medications more than three times per day or using nebulised bronchodilators or long-term oxygen therapy as well as participants unable to perform a satisfactory spirometry test were excluded from the study. An attempt was made to select non-working, non-smoking patients living in a non-smoking household to eliminate potential confounding by occupational exposures to airborne particles and by environmental tobacco smoke. The same screening questionnaire was used across the four centres to ascertain eligibility. However, each centre was allowed to choose the optimal subject recruitment method. Specifically, in Amsterdam, the panelists were recruited through distribution of 10,000 information letters accompanied by screening questionnaires. Inclusion criteria were checked using the returned screening questionnaires followed by participants’ homes’ visits. In Athens, subjects recruited through local hospitals and pulmonary chest physicians were visited at home by a pulmonologist (A.K.) and one of the investigators of the exposure assessment team (I.K.) who checked whether inclusion criteria were met. In Finland, subjects were selected from the Helsinki Metropolitan Area (including cities of Helsinki, Espoo and Vantaa) by placing advertisement on two issues of the respiratory patient association magazine (circulation ~3500 households) and notice boards of pulmonary disease clinics of four major hospitals within the study area. Candidate subjects were interviewed and screened by telephone and invited to an information session when they met the criteria. In the United Kingdom, potential study subjects living in the greater area of Birmingham were selected from the Clinic for Respiratory illnesses (CRI) database of respiratory patients at the Heartlands Hospital. Privacy regulations restricted the selections to only those that had given their written consent to be approached for research studies.

Medical ethical clearance was acquired from the relevant local medical ethics committees in all centres before the start of the recruitment. Written informed consent was obtained from each subject.

### Symptom diary

The diary was based upon diaries used in previous studies of acute effects of air pollution such as the PEACE study [21]. Although there is no real objective method of validating symptoms, a previous study by Hoek et al. provide evidence that symptoms, assessed with the same diary, are reflected in lung function drops [25]. Participants were instructed to complete a daily record about respiratory symptoms and medication taken “as needed” for six months, grading shortness of breath, wheeze, cough, phlegm and woken with breathing problems as absent (0), slight (1), or moderate/severe (2). In addition, they were asked about any limitation in performing daily life activities categorized as vigorous (such as running, lifting heavy objects, participating in strenuous sports), moderate (such as moving a table, pushing a vacuum cleaner, bowling or playing golf), walking one block/climbing one flight of stairs and leaving one’s home, because of breathing problems. This limitation could be reported in three grades: no limitation (0), yes, did activity slowly (1) and yes, avoided activity completely (2). Questions on whether they have been outside the house or town and for how long have also been included.

During the study period there was personal contact with the participants once a month to collect the completed diary forms, discuss potential problems and keep the motivation at a good level.

### Air pollution exposure

Exposure assessment has been described in previous publications [16-18,20]. In brief, during the entire study period (October 2002 to March 2004) in each city measurements of PM_2.5_, PM_10_ and PNC were performed continuously at a central site representing urban background levels [17]. The same type of condensation particle counter (TSI 3022A, TSI Inc., St. Paul, MN, USA) was used in each city to monitor PNC. 24-hour average particle mass concentration was measured with Harvard impactors for PM_2.5_ and PM_10_. Coarse particles concentrations were calculated by subtracting PM_2.5_ from PM_10_. After weighing, the absorbance of the PM_2.5_ filters (a good surrogate for elemental carbon/soot) was determined using reflectometry. PNC was transformed to “noon-to-noon” 24-hour means to coincide with the PM_2.5_ measurements. Data on concentrations of other air pollutants (ozone, nitrogen dioxide) and meteorology (air temperature, relative humidity) were collected from existing national monitoring networks in each country. We did not replace missing values in exposures variables by imputation.

### Confounder data

Time trend in health endpoints (e.g. fatigue in reporting), weather (outdoor temperature, relative humidity), medication use and day of the week were taken into account as potential confounders. Because of the staged entry of participants, we evaluated two time variables: calendar date (proxy for unmeasured confounders) and day of study for a specific subject (possibly related to fatigue).

### Quality assurance/quality control

Air pollution and health measurements were performed according to standard operating procedures (SOPs). A training workshop was organized before the start of the fieldwork and site visits were implemented during the fieldwork to identify any deviations from SOPs.

### Statistical analysis

Data analysis was done according to a predefined analysis plan. The symptom variables, initially coded as 0 for no symptoms (absent), 1 for slight symptoms and 2 for moderate/severe symptoms, were dichotomised for the analysis by setting 0 for no symptoms and 1 for slight to moderate/severe symptoms. Each symptom was analysed separately either as prevalent (irrespective of its occurrence on the previous day) or incident (when that symptom was reported to be absent on the previous day). Medication use was coded as 0 (no medication) versus 1 (intake of one or more doses) independently of the initial medication group. Every person was included in the analysis regardless of how many diary entries were made. Moreover, diary entries were excluded when participants had left the study area during the measurement period. For every pollutant the following lags were evaluated: lag 0, 1, 2 and the average of lag 0–6 days. Lag 0 was defined as the 24-hour period starting from noon of the calendar day before the health response.

A hierarchical modelling approach was used. First, regression models were fitted in each city separately to allow specific control for seasonal effects, weather and other potential confounders. Results of the individual city analysis were used in a second stage analysis (meta-analysis) to provide overall estimates [26]. We computed both fixed and random effects combined estimates. Furthermore, a chi-square test of heterogeneity of the four city-specific estimates was computed.

We applied logistic regression to obtain centre-specific effect estimates. A smooth function (natural splines with 6 degrees of freedom per year) of time was used to remove the seasonal patterns and long time trends from the data. Afterwards, same-day (lag 0) and previous-day (lag 1) mean daily temperatures were introduced simultaneously into the model. For both lags of temperature, a linear term was compared with a smoothed function (natural splines) with 2, 3 and 4 degrees of freedom and the model with the lowest Akaike’s Information Criterion (AIC) was selected. A linear term of relative humidity (lag 0) was added to the model as another indicator of weather. Finally, indicator variables for day of the week, medication use and individual differences in frequency of symptoms, were added to the model. After setting up the baseline model, the effects of the various lags of the pollutants were evaluated.

In the city specific analysis we fitted fixed effects models, described above, as well as random intercept logistic regression models using “glmmPQL” function from MASS library in R software, to take into account the correlation among each subject’s measurements. Results from the random effects analysis were very similar to those derived from fixed effects. In a few cases though, we faced convergence issues. This was even more the case when we tested a first order autoregressive correlation structure. The significance of the associations was similar between random intercept models and the models incorporating an autoregressive term.

Because of the heterogeneity of the study population, we repeated the analysis (for all air pollution measures) for the subgroup of asthmatic patients. There were not enough COPD patients to analyse these patients separately. We also fitted two pollutant models by including simultaneously PM_2.5_ and PM_10-2.5_ in order to better characterize which of the two components of PM_10_ (PM_10-2.5_ or PM_2.5_) was responsible for the observed health effects.

Effect estimates are expressed as odds ratios (OR) for an increase of 10 μg/m^3^ in PM_10_, 10 μg/m^3^ in PM_2.5_, 10 μg/m^3^ in PM_10-2.5_, 10,000 particles/cm^3^ for PNC and 1·10^-5^ m^-1^ for absorbance, in order to be comparable with other studies. For gaseous pollutants the effect estimates are expressed as OR for an increase of 10 μg/m^3^ in ozone and NO_2_ concentrations.All analyses were performed using R software [27].

## Results

### Panel characteristics

A brief description of the study population is presented in Table 1. Mean age and age range were about the same in all cities. Three participants in Athens were slightly below of the recruitment criterion of ≥35 years. In Amsterdam a large group was reported to have CNSLD. Medication use was high in the panels. Seventy seven per cent of the participants (77%) used reliever medication. Use of “as needed medication” was recorded in 26.5% of total person days in Helsinki, 13.9% in Athens, 37.9% in Amsterdam and 59.7% in Birmingham. Twenty-nine participants (21%) worked outside their home especially from Amsterdam and Birmingham. Those who worked outside their home, worked on average 19 h/week.

**Table 1:** Characteristics of four European panels of asthmatic/COPD patients

	**Helsinki**		**Athens**		**Amsterdam**		**Birmingham**	
	**n = 36**^**a**^		**n = 35**^**a**^		**n = 36**^**a**^		**n = 29**^**a**^	
Male /Female	6 / 30		19 / 16		10 / 26		7 / 22	
Asthma	3 / 28		6 / 13		3 / 8		6 / 21	
COPD	3 / 1		12 / 3		2 / 7		1 / 0	
Other^b^	0 / 1		1 / 0		5 / 11		0 / 1	
Age^c^	63.5	[36–85]	62.2	[33–84]	63.3	[46–77]	60.1	[37–76]
Asthma	62.9	[36–85]	55.7	[33–77]	62.8	[46–77]	59.6	[37–76]
COPD	65.0	[57–74]	68.7	[45–84]	63.7	[59–72]	53.0	-
Other^b^	75.0	-	78.0	-	63.2	[46–77]	69.0	-
Asthma	31	(86%)	19	(54%)	11	(31%)	27	(93%)
COPD	4	(11%)	15	(43%)	9	(25%)	1	(3.5%)
Asthma + COPD	1	(3%)	1	(3%)	4	(11%)	1	(3.5%)
CNSLD^d^	0	(0%)	0	(0%)	12	(33%)	0	(0%)
**Smoking status**								
Never smoker	26	(72%)	15	(43%)	13	(36%)	15	(52%)
Current	0	(0%)	1	(3%)	0	(0%)	3	(10%)
Ex-smoker	10	(28%)	19	(54%)	23	(64%)	11	(48%)
ETS^e^ exposure at home	0	(0%)	5	(14.7%)	0	(0%)	1	(3.4%)
**Medication use**								
Short acting β2-agonist	24	(67%)	9	(26%)	16	(44%)	28	(97%)
Reliever medication^f^	29	(81%)	21	(62%)	25	(69%)	29	(100%)
Inhaled glucocorticosteroids	34	(94%)	28	(82%)	27	(75%)	24	(83%)
Oral glucocorticosteroids	5	(14%)	5	(15%)	6	(17%)	6	(21%)
**On need medication use**								
Short acting β2-agonist	18	(50%)	8	(24%)	14	(39%)	28	(97%)
Reliever medication^f^	22	(61%)	21	(62%)	18	(50%)	29	(100%)
Inhaled glucocorticosteroids	6	(17%)	18	(53%)	7	(19%)	5	(17%)
Oral glucocorticosteroids	3	(8%)	5	(15%)	4	(11%)	5	(17%)

^a^ Total participants in panel.

^b^ Asthma + COPD or chronic non-specific lung disease.

^c^ Given as mean and [range].

^d^ Chronic non-specific lung disease.

^e^ Environmental tobacco smoke.

^f^ Includes short acting β2-agonist, long acting β2-agonist, anticholinergic drugs and combination of an anticholinergic drug and a β2-agonist.

### Symptoms

In total between 4,760 and 6,003 person days were available for analysis in the four cities. In Amsterdam, Athens and Birmingham participants filled out the diary from October 2002 to March 2004 whilst in Helsinki between October 2002 and February 2004. Missing values (person days) ranged between 9.4-15.1% in Amsterdam, 4.7-5.5% in Athens, 8.7-8.8% in Birmingham and 8.6-12.1% in Helsinki. Consistent with the composition of the panel, fairly high symptom prevalence occurred during the study period. Person days with severe symptoms were low, except for cough and phlegm. There were small differences between the cities (Table 2).

**Table 2:** Person days with symptoms in the diary (n = number of expected person days)

	**Helsinki**		**Athens**		**Amsterdam**		**Birmingham**	
	**n = 6480**	**%**	**n = 6300**	**%**	**n = 6480**	**%**	**n = 5220**	**%**
**Woken with breathing problems**								
No	4759	73.4	5334	84.7	4897	75.6	3953	75.7
Yes	978	15.1	667	10.6	958	14.8	808	15.5
Non response	743	11.5	299	4.7	625	9.6	459	8.8
**Shortness of breath**								
No	3987	61.5	4891	77.7	3410	52.6	3111	59.6
Slight symptoms	1774	27.4	1015	16.1	2179	33.6	1443	27.6
Severe symptoms	140	2.2	96	1.5	284	4.4	206	4.0
Non response	579	8.9	298	4.7	607	9.4	460	8.8
**Wheeze**								
No	4932	76.1	4325	68.7	4554	70.3	3182	61.0
Slight symptoms	923	14.2	1627	25.8	1102	17.0	1383	26.5
Severe symptoms	49	0.8	51	0.8	134	2.1	196	3.7
Non response	576	8.9	297	4.7	690	10.6	459	8.8
**Cough**								
No	3418	52.8	4303	68.3	3251	50.1	2189	41.9
Slight symptoms	2305	35.6	1583	25.1	2234	34.5	2026	38.8
Severe symptoms	184	2.8	116	1.9	289	4.5	546	10.5
Non response	573	8.8	298	4.7	706	10.9	459	8.8
**Phlegm**								
No	1973	30.5	3291	52.2	2957	45.6	2087	40.0
Slight symptoms	3462	53.4	2448	38.9	2597	40.1	2137	40.9
Severe symptoms	485	7.5	264	4.2	206	3.2	537	10.3
Non response	560	8.6	297	4.7	720	11.1	459	8.8
**Limitation of vigorous activities**^**a**^								
No	3837	59.2	2716	43.1	3714	57.3	2663	51.0
Did activity slowly	1259	19.4	2661	42.2	897	13.8	988	18.9
Avoided activity completely	603	9.3	587	9.3	891	13.8	1113	21.3
Non response	781	12.1	336	5.4	978	15.1	456	8.8
**Limitation of moderate activities**^**a**^								
No	3775	58.3	3928	62.3	4237	65.4	3420	65.5
Did activity slowly	2030	31.3	1874	29.8	1266	19.5	1211	23.2
Avoided activity completely	88	1.4	184	2.9	135	2.1	131	2.5
Non response	587	12.0	314	5.0	842	13.0	458	8.8
**Limitation of walking**^**a**^								
No	4867	75.1	3455	54.8	4449	68.7	3472	66.5
Did activity slowly	795	12.3	2438	38.7	1132	17.5	1228	23.5
Avoided activity completely	94	1.4	62	1.0	152	2.3	63	1.2
Non response	724	11.2	345	5.5	747	11.5	457	8.8

^a^ due to breathing problems.

### Air pollution concentrations

Helsinki had the lowest median concentrations for all PM components whilst Athens had the highest. However, maximum concentrations of PM_2.5_ were observed in Amsterdam (103.4 μg/m^3^) and of PM_10-2.5_ (152.6 μg/m^3^) in Helsinki (Table 3).

**Table 3:** Daily (24 hours noon-to-noon, central site) median air pollution concentration and meteorology in the four cities

		**Helsinki**			**Athens**			**Amsterdam**			**Birmingham**		
		**10/2002-4/2004**			**10/2002-3/2004**			**10/2002-3/2004**			**11/2002-3/2004**		
		**% missing**	**Median**	**Range**	**% missing**	**Median**	**Range**	**% missing**	**Median**	**Range**	**% missing**	**Median**	**Range**
PNC	10^4^·cm^-3^	5.0	1.3	(0.2, 4.4)	10.5	2.0	(0.3, 6.6)	2.4	1.8	(0.8, 4.4)	21.2	1.9	(0.2, 5.1)
PM_10_	μg·m^-3^	38.1	12.4	(0.2, 156.4)	6.3	51.7	(8.5, 158.7)	3.3	26.6	(7.4, 126.0)	14.6	16.6	(2.8, 126.2)
PM_2.5_	μg·m^-3^	34.6	7.4	(0.3, 33.2)	6.3	22.7	(2.4, 79.1)	3.1	16.7	(4.0, 103.4)	12.8	8.4	(0.7, 71.9)
PM_10-2.5_	μg·m^-3^	39.1	4.6	(0.0, 152.6)	6.7	28.8	(0.7, 126.4)	3.7	9.4	(0.9, 24.2)	15.6	6.9	(0.3, 118.9)
Absorbance	10^-5^·m^-1^	38.9	1.2	(0.2, 3.8)	6.3	3.5	(0.9, 8.4)	3.1	1.9	(0.5, 7.2)	12.8	1.3	(0.2, 4.9)
NO_2_	μg·m^-3^	1.4	22.7	(4.5, 77.9)	27.4	39.9	(11.8, 110.9)	0.4	38.4	(10.4, 97.3)	0.2	34.4	(7.3, 83.3)
Ozone	μg·m^-3^	1.7	42.5	(4.1, 93.2)	11.8	46.9	(4.7, 108.2)	9.4	33.1	(0.9, 104.3)	0.0	37.3	(0.9, 106.6)
Temperature	°C	0.0	2.0	(−22.8, 25.6)	0.0	15.0	(−3.1, 33.2)	0.0	9.1	(−6.1, 25.3)	0.0	9.2	(−1.4, 26.9)
Rel. humidity	%	0.0	80.7	(36.5, 100.0)	0.0	66.1	(21.8, 93.2)	0.0	80.8	(38.5, 98.7)	0.0	79.3	(45.8, 97.9)

### Air pollution effects on symptoms-limitation in activities due to breathing problems

#### Prevalence analyses

We observed very small differences in fixed and random effects combined estimates. In Tables 4 and 5 combined odds ratios for the association of particulate matter indices, NO_2_, ozone and prevalence of symptoms and limitation in activities are presented, using random effects models adjusting for the above mentioned confounders and “as needed” medication. When all participants were included in the analysis as a total, we found that a 10 μg/m^3^ increase in PM_10_ was significantly associated at the nominal level with shortness of breath in the lag 1 whilst the association in the lags 2 and 0 to 6 was of borderline significance. However, none of the associations was significant for the asthma group. Significant association was also observed for wheezing and limitation in walking due to breathing problems (lag 1). The association was driven by the PM_10-2.5_ component of PM_10_ and much less by PM_2.5_. Coarse particles concentrations were positively associated with most symptom and restriction of activities variables in lag1. In addition, the modest correlations between PM_10-2.5_ and PM_2.5_ (0.08, 0.40, 0.35 and 0.13 for Amsterdam, Athens, Birmingham and Helsinki respectively) did allow us to apply a two-pollutant model in order to separate and further evaluate the effects of the two components of PM_10_. The magnitude of the associations for PM_10-2.5_ with prevalence of symptoms and restriction of activities remained approximately the same or increased when we applied a two-pollutant model with PM_2.5_ (Table 6).

**Table 4:** **Associations of particulate matter indices, NO**_**2 **_**and O**_**3 **_**with prevalence of symptoms in all participants and the subgroup of asthmatics (random effects pooled estimates)**

**Symptom**	**Pollutant**	** Lag0**		** Lag1**		** Lag2**		** Lag06**	
		**OR**	**95% CI**	**OR**	**95% CI**	**OR**	**95% CI**	**OR**	**95% CI**
**Woken with breathing problems**									
	PM_10_								
Total		1.001	0.966-1.037	1.010	0.964-1.059	0.978	0.928-1.030	1.009	0.881-1.155
Asthmatics		0.977	0.937-1.019	0.982	0.923-1.044	0.953	0.881-1.031	0.947	0.855-1.049
	PM_2.5_								
Total		0.997	0.952-1.044	0.980	0.915-1.049	0.953	0.886-1.025	0.889	0.682-1.160
Asthmatics		0.988	0.932-1.048	0.955	0.863-1.057	0.944	0.868-1.026	0.943	0.787-1.130
	PM_10 - 2.5_								
Total		1.020	0.883-1.179	1.047	0.989-1.109	0.996	0.935-1.062	1.019	0.860-1.208
Asthmatics		0.959	0.797-1.154	1.009	0.952-1.070	0.915	0.731-1.147	0.689	0.381-1.247
	PNC								
Total		0.971	0.865-1.090	1.027	0.952-1.109	0.958	0.863-1.064	0.910	0.638-1.298
Asthmatics		1.012	0.844-1.212	1.047	0.961-1.141	1.019	0.939-1.106	1.195	0.953-1.497
	Absorbance								
Total		1.014	0.952-1.079	1.018	0.966-1.073	0.971	0.922-1.022	0.929	0.777-1.111
Asthmatics		1.051	0.874-1.264	1.026	0.940-1.120	0.978	0.916-1.044	0.967	0.805-1.162
	NO_2_								
Total		0.980	0.940-1.021	0.983	0.943-1.026	0.970	0.926-1.016	0.969	0.856-1.098
Asthmatics		1.017	0.918-1.126	0.995	0.935-1.059	0.964	0.918-1.012	0.984	0.850-1.140
	O_3_								
Total		**1.063**	**1.020-1.108**	1.023	0.957-1.094	1.010	0.959-1.064	1.037	0.896-1.200
Asthmatics		0.982	0.896-1.077	1.001	0.925-1.082	1.009	0.926-1.099	1.075	0.935-1.235
**Shortness of breath**									
	PM_10_								
Total		0.998	0.970-1.026	**1.037**	**1.002-1.074**	1.014	0.986-1.042	1.050	0.998-1.106
Asthmatics		0.992	0.956-1.028	1.030	0.994-1.067	0.997	0.962-1.032	1.049	0.936-1.176
	PM_2.5_								
Total		1.001	0.942-1.063	1.035	0.974-1.099	1.026	0.984-1.070	1.027	0.944-1.117
Asthmatics		1.006	0.951-1.063	1.032	0.977-1.091	1.018	0.965-1.074	1.005	0.828-1.219
	PM_10 - 2.5_								
Total		0.995	0.949-1.042	**1.060**	**1.015-1.107**	1.002	0.949-1.057	1.044	0.947-1.151
Asthmatics		0.972	0.915-1.031	1.028	0.973-1.086	0.978	0.925-1.033	1.010	0.872-1.168
	PNC								
Total		0.972	0.901-1.048	**0.910**	**0.844-0.982**	**0.919**	**0.860-0.982**	0.908	0.770-1.071
Asthmatics		0.976	0.898-1.062	0.925	0.817-1.046	0.952	0.879-1.031	1.032	0.860-1.237
	Absorbance								
Total		1.019	0.936-1.109	1.042	0.954-1.138	1.026	0.978-1.077	1.064	0.929-1.218
Asthmatics		1.014	0.930-1.106	1.055	0.975-1.143	1.048	0.985-1.116	1.218	0.950-1.562
	NO_2_								
Total		1.011	0.934-1.094	0.996	0.915-1.085	0.985	0.940-1.032	1.011	0.874-1.170
Asthmatics		0.991	0.886-1.108	0.987	0.880-1.106	0.984	0.915-1.058	1.013	0.846-1.213
	O_3_								
Total		0.988	0.957-1.021	0.961	0.921-1.003	0.975	0.946-1.004	0.962	0.914-1.014
Asthmatics		0.967	0.931-1.004	**0.932**	**0.898-0.967**	**0.932**	**0.888-0.979**	0.915	0.828-1.011
**Wheezing**									
	PM_10_								
Total		1.026	0.980-1.074	**1.027**	**1.000-1.055**	1.011	0.981-1.041	0.989	0.869-1.125
Asthmatics		1.012	0.955-1.073	1.011	0.979-1.043	0.994	0.964-1.026	0.984	0.894-1.082
	PM_2.5_								
Total		0.998	0.925-1.077	1.004	0.931-1.082	0.982	0.882-1.094	0.873	0.629-1.213
Asthmatics		0.971	0.886-1.064	0.966	0.888-1.050	0.973	0.886-1.067	0.902	0.681-1.195
	PM_10 - 2.5_								
Total		1.041	0.990-1.094	**1.073**	**1.028-1.120**	1.023	0.980-1.068	1.053	0.966-1.147
Asthmatics		1.045	0.972-1.124	1.044	0.995-1.096	1.003	0.957-1.051	1.008	0.909-1.119
	PNC								
Total		0.934	0.791-1.104	0.947	0.816-1.099	0.985	0.841-1.154	1.092	0.639-1.865
Asthmatics		0.975	0.815-1.165	0.989	0.821-1.191	1.046	0.842-1.301	1.406	0.730-2.705
	Absorbance								
Total		0.980	0.910-1.055	1.000	0.930-1.075	0.999	0.888-1.123	0.926	0.644-1.332
Asthmatics		0.970	0.890-1.058	0.984	0.906-1.070	0.975	0.853-1.113	1.008	0.755-1.347
	NO_2_								
Total		0.995	0.922-1.074	0.983	0.923-1.047	1.003	0.933-1.078	1.004	0.828-1.217
Asthmatics		0.995	0.934-1.060	0.980	0.916-1.048	0.986	0.895-1.086	0.999	0.838-1.191
	O_3_								
Total		1.008	0.966-1.051	1.012	0.965-1.061	1.009	0.949-1.073	1.031	0.933-1.138
Asthmatics		0.985	0.948-1.025	0.998	0.959-1.038	1.046	0.947-1.156	1.060	0.916-1.228
**Cough**									
	PM_10_								
Total		1.001	0.975-1.027	1.014	0.985-1.045	0.999	0.957-1.043	1.007	0.913-1.110
Asthmatics		0.993	0.952-1.034	0.991	0.956-1.026	0.989	0.935-1.047	0.969	0.900-1.043
	PM_2.5_								
Total		**0.960**	**0.922-0.999**	0.971	0.933-1.011	0.962	0.919-1.008	0.901	0.753-1.079
Asthmatics		0.940	0.847-1.042	0.949	0.884-1.019	0.946	0.879-1.019	0.918	0.761-1.107
	PM_10 - 2.5_								
Total		1.099	0.943-1.282	1.089	0.956-1.240	1.043	0.958-1.137	1.210	0.772-1.896
Asthmatics		1.016	0.966-1.069	1.024	0.974-1.076	1.003	0.912-1.103	1.005	0.902-1.120
	PNC								
Total		0.981	0.916-1.051	1.009	0.944-1.079	0.968	0.895-1.047	0.894	0.714-1.119
Asthmatics		0.979	0.906-1.058	0.972	0.900-1.050	0.918	0.807-1.044	0.824	0.618-1.098
	Absorbance								
Total		**0.939**	**0.898-0.982**	0.976	0.932-1.022	0.959	0.917-1.003	1.083	0.797-1.472
Asthmatics		0.937	0.833-1.054	0.976	0.892-1.068	**0.942**	**0.891-0.997**	1.078	0.782-1.486
	NO_2_								
Total		0.971	0.937-1.007	0.981	0.945-1.017	0.965	0.931-1.000	0.959	0.899-1.024
Asthmaticss		0.980	0.935-1.026	0.986	0.941-1.033	0.972	0.925-1.020	0.981	0.903-1.066
	O_3_								
Total		**1.061**	**1.013-1.111**	**1.049**	**1.016-1.083**	**1.059**	**1.027-1.091**	1.066	0.982-1.157
Asthmatics		**1.062**	**1.016-1.110**	**1.051**	**1.002-1.102**	**1.058**	**1.022-1.095**	1.106	0.939-1.302

Bold are significant pooled effects.

**Table 5:** **Associations of particulate matter indices, NO**_**2 **_**and O**_**3 **_**with limitation in activities due to breathing problems in all participants and the subgroup of asthmatics (random effects pooled estimates)**

**Symptom**	**Pollutant**	**Lag0**		**Lag1**		**Lag2**		**Lag06**	
		**OR**	**95% CI**	**OR**	**95% CI**	**OR**	**95% CI**	**OR**	**95% CI**
**Vigorous activities**									
	PM_10_								
Total		1.018	0.950-1.092	1.028	0.957-1.103	1.026	0.956-1.102	0.989	0.854-1.146
Asthmatics		0.987	0.869-1.121	1.006	0.873-1.158	1.010	0.873-1.168	0.924	0.726-1.177
	PM_2.5_								
Total		1.016	0.910-1.135	1.016	0.892-1.158	1.039	0.922-1.169	1.005	0.831-1.216
Asthmatics		0.992	0.856-1.150	0.995	0.843-1.174	1.019	0.858-1.211	0.944	0.743-1.198
	PM_10 - 2.5_								
Total		1.093	0.933-1.281	1.114	0.949-1.308	1.049	0.948-1.160	1.230	0.851-1.779
Asthmatics		0.980	0.817-1.175	1.005	0.816-1.238	1.019	0.919-1.130	0.956	0.612-1.494
	PNC								
Total		1.001	0.888-1.129	1.018	0.916-1.133	1.054	0.985-1.129	0.906	0.778-1.054
Asthmatics		0.979	0.895-1.070	1.014	0.924-1.112	1.065	0.976-1.163	0.927	0.748-1.149
	Absorbance								
Total		0.999	0.894-1.117	1.021	0.917-1.137	1.010	0.885-1.152	0.994	0.691-1.432
Asthmatics		1.031	0.924-1.151	1.063	0.938-1.206	1.069	0.919-1.243	0.984	0.643-1.507
	NO_2_								
Total		0.988	0.940-1.038	1.010	0.971-1.051	1.037	0.979-1.097	1.007	0.844-1.202
Asthmatics		0.995	0.916-1.081	1.028	0.960-1.100	1.046	0.945-1.158	0.942	0.713-1.245
	O_3_								
Total		1.033	0.935-1.141	1.031	0.946-1.124	1.024	0.932-1.125	1.107	0.879-1.394
Asthmatics		0.980	0.887-1.083	0.988	0.927-1.053	0.979	0.922-1.040	0.981	0.850-1.133
**Moderate activities**									
	PM_10_								
Total		0.973	0.888-1.067	0.973	0.923-1.026	0.973	0.907-1.044	0.904	0.729-1.122
Asthmatics		0.946	0.818-1.094	0.958	0.857-1.071	0.979	0.897-1.069	0.859	0.680-1.086
	PM_2.5_								
Total		0.922	0.749-1.136	0.950	0.848-1.065	0.963	0.862-1.077	0.953	0.808-1.124
Asthmatics		0.896	0.712-1.126	0.979	0.839-1.142	0.965	0.846-1.102	0.938	0.762-1.154
	PM_10 - 2.5_								
Total		1.068	0.913-1.249	1.023	0.932-1.122	1.014	0.966-1.064	0.912	0.667-1.248
Asthmatics		1.000	0.943-1.062	0.987	0.932-1.045	1.007	0.955-1.062	0.727	0.411-1.289
	PNC								
Total		1.077	0.937-1.239	1.034	0.899-1.189	1.010	0.927-1.100	0.935	0.762-1.146
Asthmatics		1.075	0.977-1.183	1.025	0.922-1.139	1.016	0.873-1.184	0.835	0.680-1.025
	Absorbance								
Total		0.973	0.891-1.062	0.973	0.869-1.088	0.974	0.851-1.116	1.036	0.740-1.451
Asthmatics		0.994	0.871-1.135	0.993	0.854-1.155	0.984	0.814-1.190	0.985	0.618-1.570
	NO_2_								
Total		1.004	0.917-1.100	0.991	0.915-1.074	1.007	0.932-1.088	1.064	0.873-1.296
Asthmatics		1.004	0.897-1.123	0.988	0.886-1.100	1.000	0.889-1.125	0.999	0.718-1.390
	O_3_								
Total		0.970	0.899-1.046	1.000	0.916-1.091	1.002	0.939-1.070	1.022	0.879-1.189
Asthmatics		0.940	0.874-1.012	0.979	0.901-1.064	0.979	0.921-1.041	1.007	0.812-1.250
**Walking**									
	PM_10_								
Total		1.010	0.924-1.104	**1.039**	**1.007-1.073**	1.012	0.976-1.049	1.074	0.966-1.194
Asthmatics		1.009	0.921-1.105	**1.055**	**1.018-1.094**	1.023	0.987-1.061	1.009	0.823-1.237
	PM_2.5_								
Total		1.000	0.917-1.091	1.019	0.953-1.088	0.963	0.850-1.090	0.839	0.574-1.225
Asthmatics		1.008	0.882-1.152	**1.097**	**1.032-1.167**	**1.078**	**1.013-1.147**	0.934	0.663-1.316
	PM_10 - 2.5_								
Total		1.072	0.904-1.273	**1.076**	**1.026-1.128**	1.044	0.997-1.092	1.079	0.819-1.420
Asthmatics		1.012	0.884-1.159	**1.060**	**1.005-1.119**	1.013	0.959-1.069	0.911	0.554-1.499
	PNC								
Total		0.978	0.797-1.199	0.986	0.915-1.063	1.007	0.937-1.083	0.975	0.670-1.418
Asthmatics		0.906	0.798-1.029	1.010	0.921-1.106	1.013	0.928-1.106	0.804	**0.658-0.981**
	Absorbance								
Total		1.014	0.915-1.124	1.038	0.956-1.128	1.013	0.942-1.089	0.852	0.544-1.333
Asthmatics		1.036	0.939-1.143	**1.100**	**1.031-1.174**	1.078	0.965-1.204	0.942	0.610-1.456
	NO_2_								
Total		0.979	0.940-1.019	1.011	0.959-1.065	1.034	0.993-1.077	1.075	0.931-1.241
Asthmatics		0.988	0.924-1.057	1.048	0.967-1.136	1.076	0.968-1.196	1.093	0.886-1.349
	O_3_								
Total		1.012	0.975-1.050	1.004	0.961-1.048	1.020	0.983-1.059	1.038	0.971-1.109
Asthmatics		0.999	0.948-1.053	0.982	0.933-1.033	1.003	0.939-1.071	1.048	0.913-1.203

Bold are significant pooled effects.

**Table 6:** **Associations of PM**_**10-2.5 **_**and PM**_**2.5 **_**with prevalence of symptoms and limitation in activities due to breathing problems after applying two pollutant models (random effects pooled estimates)**

**Symptom**	**Lag0**		**Lag1**		**Lag2**		**Lag06**	
	**OR**	**95% CI**	**OR**	**95% CI**	**OR**	**95% CI**	**OR**	**95% CI**
**Woken with breathing problems**								
PM_10 - 2.5_	1.029	0.877-1.208	**1.060**	**1.006-1.117**	1.017	0.957-1.080	1.118	0.846-1.477
PM_2.5_	0.999	0.951-1.048	0.969	0.909-1.032	0.939	0.872-1.011	0.751	0.403-1.400
**Shortness of breath**								
PM_10 - 2.5_	0.985	0.937-1.037	1.047	0.999-1.096	0.993	0.931-1.059	1.042	0.938-1.156
PM_2.5_	1.006	0.945-1.070	1.025	0.977-1.076	1.024	0.980-1.070	1.042	0.960-1.131
**Wheezing**								
PM_10 - 2.5_	**1.063**	**1.016-1.112**	**1.078**	**1.002-1.159**	1.031	0.959-1.109	**1.107**	**1.010-1.213**
PM_2.5_	0.982	0.895-1.078	0.958	0.854-1.075	0.962	0.850-1.089	0.921	0.721-1.177
**Cough**								
PM_10 - 2.5_	1.140	0.950-1.367	1.127	0.955-1.329	1.060	0.977-1.150	1.293	0.885-1.890
PM_2.5_	**0.945**	**0.905-0.987**	**0.954**	**0.914-0.995**	0.946	0.893-1.001	0.900	0.779-1.040
**Vigorous activities**								
PM_10 - 2.5_	1.113	0.894-1.386	1.100	0.923-1.311	1.047	0.903-1.214	1.248	0.839-1.854
PM_2.5_	1.009	0.890-1.144	1.006	0.862-1.175	1.047	0.918-1.193	0.939	0.813-1.084
**Moderate activities**								
PM_10 - 2.5_	1.097	0.891-1.350	1.036	0.917-1.170	1.006	0.956-1.059	0.965	0.699-1.333
PM_2.5_	0.919	0.748-1.129	0.950	0.844-1.070	0.967	0.873-1.070	0.937	0.797-1.103
**Walking**								
PM_10 - 2.5_	1.090	0.900-1.321	**1.073**	**1.020-1.128**	1.047	0.990-1.107	1.128	0.946-1.344
PM_2.5_	0.998	0.950-1.049	1.001	0.937-1.071	0.969	0.862-1.088	0.790	0.466-1.339

Bold are significant pooled effects.

The above-mentioned positive associations with PM_10-2.5_ (Tables 4 and 5) were reduced and no longer significant after restricting the analysis to the asthmatic only participants. A significant association remained with restricting walking activities and wheeze (borderline).

Ozone was significantly associated with cough at lag 0, lag 1, lag 2 and with woken with breathing problems at lag 0. Furthermore, the associations with wheezing, limitation in vigorous activities and walking due to breathing problems remained positive across all examined lags although, non significant. Negative but non significant associations were observed with shortness of breath across all examined lags. However, a significant preventive effect of ozone for shortness of breath was revealed for lags 1 and 2, in the asthma group. Moreover, in the asthmatics, negative associations were also observed for ozone with woken with breathing problems (lag 0), wheezing (lag 0 and lag 1), and with limitation in activities due to breathing problems (most of the lags), although non significant (Tables 4 and 5).

Neither PM_2.5_ nor NO_2_ were consistently associated with any symptom or limitation in activities variable. As for PNC a (mostly non-significant) negative association was observed with most symptoms whilst the positive associations with woken with breathing problems and cough in lag 1 as well as in limitation of activities due to breathing problems (mainly vigorous and moderate) in lags 0, 1, 2 did not reach the nominal level of significance. Moreover, for PNC a change of the negative associations with woken with breathing problems towards positive values, across all lags, was observed when the analysis was restricted to the asthmatic participants, although non significant (Tables 4 and 5).

**Figure 1:**
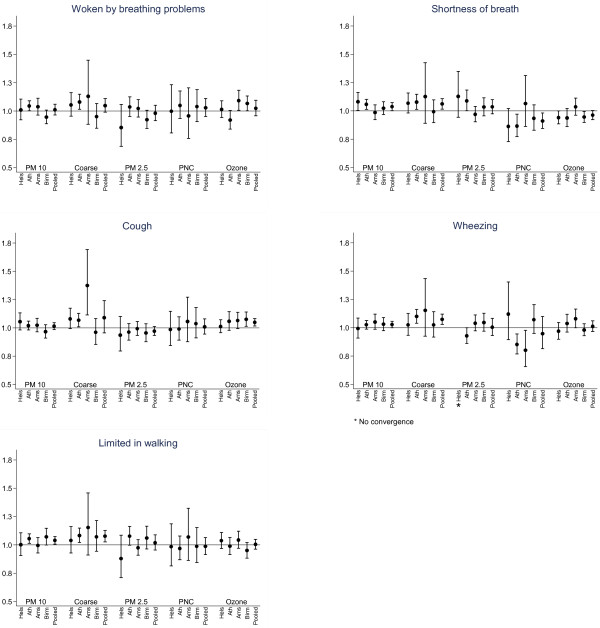
**Odds ratio (95% CI) for prevalence of symptoms and limitation in activities associated with an increase of 10 μg/m**^**3 **^**in previous day (lag1) concentrations of each pollutant (10,000/cm**^**3 **^**for PNC) in each participating city and overall estimate (random effects pooled estimates).**

Centre specific and overall effect estimates with 95 percent confidence intervals (95% CI) for the association of each symptom and air pollutant in lag1 are presented in Figure 1. Odds ratios (OR) for the effect of PM_10-2.5_ were consistently above one in almost every city as well as in the pooled data using random effects meta-analysis.

### Incidence analyses

Patterns similar to those in the combined prevalence analyses were observed for the associations of incident symptoms and particles especially the coarse fraction. Shortness of breath was consistently associated with PM_10_ and PM_10-2.5_ in lag 1 with no indication of heterogeneity between the centres (OR = 1.045, 95% CI: 1.008, 1.083 and OR = 1.065, 95% CI: 1.009, 1.124 respectively). There was also a tendency towards positive associations between PM_10-2.5_ and incidence of wheezing, cough and limitation in walking but none of the associations were statistically significant. Additionally, ozone was positively associated with cough in lags 1 and 2 as well as the average lag 0–6 days but only in lag 2 the association reached the nominal level of significance (Table 7).

**Table 7:** **Associations of particulate matter indices, NO**_**2 **_**and O**_**3 **_**with incidence of symptoms in the four panels (random effects pooled estimates)**

**Symptom**	**Pollutant**	**Lag0**		**Lag1**		**Lag2**		**Lag06**	
		**OR**	**95% CI**	**OR**	**95% CI**	**OR**	**95% CI**	**OR**	**95% CI**
**Woken with breathing problems**	PM_10_	0.994	0.951-1.038	0.992	0.950-1.037	**0.920**	**0.877-0.966**	0.925	0.852-1.004
	PM_2.5_	0.995	0.931-1.064	0.965	0.903-1.033	**0.919**	**0.857-0.984**	**0.878**	**0.784-0.983**
	PM_10 - 2.5_	0.994	0.928-1.065	1.016	0.950-1.088	**0.823**	**0.701-0.967**	0.882	0.654-1.189
	PNC	0.878	0.736-1.048	1.041	0.934-1.160	0.887	0.716-1.099	0.816	0.563-1.181
	Absorbance	1.003	0.930-1.082	0.998	0.924-1.077	0.909	0.815-1.012	0.861	0.729-1.016
	NO_2_	1.013	0.936-1.096	1.015	0.953-1.082	0.956	0.900-1.016	0.923	0.817-1.043
	O_3_	1.016	0.912-1.131	0.988	0.937-1.041	0.994	0.945-1.045	1.069	0.980-1.165
**Shortness of breath**	PM_10_	1.008	0.958-1.060	**1.045**	**1.008-1.083**	0.992	0.954-1.031	1.048	0.974-1.127
	PM_2.5_	1.019	0.963-1.079	1.039	0.982-1.098	0.999	0.941-1.061	1.069	0.976-1.171
	PM_10 - 2.5_	0.993	0.929-1.062	**1.065**	**1.009-1.124**	0.977	0.906-1.054	1.101	0.883-1.373
	PNC	0.963	0.871-1.066	**0.871**	**0.785-0.966**	0.968	0.861-1.088	0.992	0.767-1.283
	Absorbance	1.017	0.949-1.091	0.995	0.931-1.064	0.988	0.925-1.055	1.372	0.756-2.490
	NO_2_	1.022	0.970-1.076	0.969	0.918-1.022	0.998	0.947-1.052	1.036	0.921-1.165
	O_3_	0.989	0.945-1.035	0.987	0.944-1.031	1.021	0.980-1.065	1.021	0.950-1.097
**Wheezing**	PM_10_	1.009	0.963-1.057	0.997	0.903-1.100	1.000	0.930-1.076	1.025	0.838-1.254
	PM_2.5_	1.009	0.949-1.073	0.989	0.884-1.106	1.023	0.925-1.132	1.065	0.839-1.351
	PM_10 - 2.5_	1.010	0.946-1.079	1.044	0.763-1.427	0.970	0.872-1.079	1.065	0.910-1.246
	PNC	0.968	0.832-1.127	1.060	0.938-1.198	1.051	0.950-1.163	1.207	0.877-1.660
	Absorbance	0.991	0.925-1.061	1.018	0.950-1.091	1.025	0.957-1.099	1.106	0.922-1.328
	NO_2_	1.009	0.951-1.071	0.986	0.932-1.043	1.028	0.974-1.086	1.089	0.988-1.199
	O_3_	0.968	0.921-1.017	0.978	0.899-1.064	0.975	0.931-1.022	0.940	0.861-1.026
**Cough**	PM_10_	1.014	0.961-1.070	1.005	0.966-1.045	0.982	0.943-1.024	1.017	0.948-1.092
	PM_2.5_	0.976	0.891-1.069	0.969	0.912-1.030	0.990	0.932-1.052	0.991	0.898-1.094
	PM_10 - 2.5_	1.060	0.938-1.198	1.037	0.975-1.104	0.972	0.909-1.040	1.160	0.875-1.538
	PNC	0.956	0.834-1.095	1.024	0.924-1.135	0.983	0.888-1.089	1.109	0.819-1.500
	Absorbance	0.991	0.879-1.116	0.980	0.911-1.054	0.942	0.877-1.012	1.029	0.828-1.279
	NO_2_	0.994	0.942-1.049	0.968	0.884-1.061	0.975	0.923-1.030	0.999	0.844-1.182
	O_3_	0.984	0.939-1.032	1.027	0.950-1.109	**1.044**	**1.000-1.090**	1.030	0.938-1.132

Bold are significant pooled effects.

## Discussion

In this multicentre study we found consistent positive associations between coarse particles central sites concentrations and prevalence of respiratory symptoms, as recorded in a 6-month diary, in four panels of participants with predominantly mild to moderate asthma or COPD in four European cities participating in the RUPIOH study. We also found a significant association of ozone with cough and woken with breathing problems, but not with other symptoms. Neither PM_2.5_ nor NO_2_ were consistently associated with any symptom or limitation in activities variable. As for PNC a (mostly non-significant) negative association was observed with most symptoms whilst positive associations with woken with breathing problems and cough as well as with limitation of vigorous and moderate activities due to breathing problems, did not reach the nominal level of significance. Interestingly, for PNC a change of the negative associations with woken with breathing problems towards positive values, across all lags, was observed when the analysis was restricted to the asthmatic participants, although non significant at the nominal level. An analysis of the asthmatic subgroup showed generally lower odds ratios for PM_10-2.5_.

One particularity and strength of the RUPIOH study is the in depth assessment of particulate air pollution by measuring PM_10_, PM_2.5_ (then deriving coarse particles), filters absorbance as well as the number of ultrafine particles. Previous work from RUPIOH that included air pollution monitoring for one week inside and directly outside participants’ homes reported no association with lung function [19]. As the authors stated a potential explanation could be the high prevalence of medication use, the short period of measurements (one week) that limited the ability to assess lagged effects over several days or absence of an effect. The high prevalence of medication use may also have covered some associations in the present study.

A limitation of the study is the inclusion of both COPD and asthma patients. COPD and asthma are two diseases with different underlying pathophysiological mechanisms and day to day variability in their symptoms [22,23]. Mixing of the two diseases does not create bias in the analysis in the full population as we adjusted for differences in health status between individuals. The generalizability of the size of the effect estimates is more affected by the population. Though asthma and COPD are different diseases, we are not aware of studies that have demonstrated differences in the magnitude of response to air pollution. In our recently accepted paper in the same panel we did not find any difference in the effect of PM_10-2.5_ on total nitrate and nitrite concentrations in exhaled breath condensate (EBC NOx), a marker of oxidative stress between asthma and COPD patients [28]. In that study we could evaluate disease status as the outcome was a continuous variable. Unfortunately, an analysis restricted to COPD patients was not possible due to the small number of COPD patients participating in Helsinki and Birmingham. Hence, we also could not test whether the smaller PM_10-2.5_ effect in the asthmatic subgroup differed significantly from the COPD subgroup.

Our coarse particle findings are however consistent with the observation that in the RUPIOH study only the PM_10-2.5_ concentration at central sites was significantly associated with increased EBC NOx collected during the same week as the spirometry [28]. EBC NOx has been suggested as a reliable marker of oxidative stress [29-31]. The link between PM_10-2.5_ with oxidative stress and airway inflammation may explain the increase in respiratory symptoms we found.

In this study, we also report significant positive associations of ozone with cough throughout most of the examined lags both in the analysis of total participants and the subgroup of the asthmatics that are consistent with previous epidemiological and toxicological studies [32]. In addition, positive associations, but not significant in the nominal level, were observed with most of symptoms when total participants were included in the analysis. However, when we restricted the analysis to the subgroup of asthmatics, a significant preventive effect of ozone for shortness of breath was revealed for lags 1 and 2. Negative associations were also observed with woken with breathing problems, wheezing and with limitation in activities due to breathing problems, although non significant. Factors like high medication use, intrinsic differences in responsiveness to ozone among individuals, adaptation to ozone issues or other spurious effects may have been responsible for these findings [32].

In the last two decades a substantial body of literature has focused on the harmful health effects of PM_10_ and PM_2.5_[15,32]. As a result guideline values have been recommended by the U.S. Environmental Protection Agency and World Health Organization for both indicators of PM pollution to protect public health [2,3]. However, from recent studies there is increasing evidence that the health effects of coarse particles should not be underestimated. In a systematic review of epidemiological studies that have analyzed fine and coarse PM jointly, Brunekreef and Forsberg examined the epidemiological evidence for effects of coarse particles on health [15]. They concluded that the effects of PM_10-2.5_ were stronger than or as strong as PM_2.5_ on short-term respiratory morbidity. Furthermore, in a national multicity study, Zanobetti and Schwartz found a strong association of both fine and coarse particles with daily deaths in 112 U.S. cities [33]. A 10 μg/m^3^ increase in PM_10-2.5_ was significantly associated with total mortality, stroke, cardiovascular, and respiratory mortality, the latter of which showing the largest effect (a 1.2% increase). Mechanistically, these effects may be due either to biogenic factors or to metals carried by PM_10-2.5_ by activation of inflammatory and oxidative stress pathways [34-36]. The findings of our study support previous epidemiological and toxicological evidence that health effects due to the coarse fraction may be substantial [37].

The large number of calculations we have done could have given some statistically significant associations by chance. However, multiple testing is an unlikely explanation of the findings in the current study. In the full study population we found 14 significant associations of which 10 were positive; in the asthmatics subgroup we found 12 significant associations of which 8 were positive. The consistency of associations (e.g. for ozone and cough) further argues against chance as the main explanation for our findings. Additionally, in the full study population the significant associations for PM_10-2.5_ were supported by elevated though not nominally significant ORs for other lags and symptoms. Finally, ORs were mainly homogeneous across centers. Moreover, the modest correlations between PM_10-2.5_ and PM_2.5_ did allow us to apply a two-pollutant model in order to separate and further evaluate the effects of the two components of PM_10_. The magnitude of the associations for PM_10-2.5_ with prevalence of symptoms and restriction of activities remained approximately the same or increased when we applied a two-pollutant model with PM_2.5_.

The majority of studies that investigated health effects of particulate pollutants have expressed results on a mass basis. It has been suggested that when taking into consideration particle number or surface area, the pulmonary dose of toxic material related to PM_2.5_ may be much larger than the dose related to PM_10-2.5_ that for this reason alone, comparison on a mass basis may be less informative [15]. In our study we separately investigated the mass and the number effect. Neither central site PM_2.5_ nor PNC were consistently associated with symptoms. The association we observed with PM_10-2.5_, if not by chance, may also imply that a central measurement site is more appropriate for measurements of mass concentrations than for PNC. The analysis of RUPIOH data by Puustinen et al. showed generally high correlations between 24 hour average central site and residential outdoor concentrations for PM_2.5_ and soot with a lesser median correlation for PM_10_ and a lower correlation for PNC and PM_10-2.5_[17]. For PM_10-2.5_ correlations between central site and home outdoor measurements were 0.66, 0.74, 0.89 and 0.64 in Helsinki, Athens, Amsterdam and Birmingham respectively. A central site thus provides a reasonably good estimate of more local exposures even for coarse particles.

The relatively high divergence of PM_10-2.5_ concentrations between proximate sites in the UK has recently been confirmed by Liu and Harrison [38]. Consequently, for both PNC and PM_10-2.5_, there is a higher probability of exposure misclassification than for PM_2.5_ or soot. The finding of significant associations with respiratory health outcomes for PM_10-2.5_ but not for PNC is therefore quite striking but consistent with the recent findings of a time series study in London which found significant associations between PNC and cardiovascular health outcomes whilst PM mass metrics were associated with respiratory outcomes [39]. A plausible explanation could be the existence of different biological and pathophysiological mechanisms through which PM_10-2.5_ and PNC exert their adverse effects or different target organs. The results of recent toxicological studies support the theory that PM_10-2.5_ exert their effects at the site of deposition in the airways whereas PNC, after crossing the alveolar epithelial barrier, enter into the systemic circulation and affect cardiovascular function [40,41]. This theory could explain the positive associations we found between PM_10-2.5_ and respiratory symptoms.

In summary, our study contributes to the literature on the health effects of PM in respiratory patients. Moreover, the results of our study are in agreement with the findings of recent epidemiological and toxicological studies and provide enough evidence to conclude that it is prudent to keep PM_10-2.5_ regulated in addition to fine particles.

## Conclusions

Our study adds to the limited existing evidence of recent epidemiological and toxicological studies that health effects due to the coarse fraction of ambient PM may be substantial. Further studies are needed to clarify possible different effects of PM on COPD and asthmatic patients. The observed associations suggest it is prudent to regulate also coarse particles in addition to fine particles.

## Abbreviations

AIC: Akaike’s information criterion; CNSLD: Chronic non-specific lung disease; COPD: Chronic obstructive pulmonary disease; EBC NOx: Total nitrate and nitrite concentrations in exhaled breath condensate; OR: Odds ratio; PM: Particulate matter; PM_10-2.5_: Coarse particles; PM_10_: Mass concentration of particles less than 10 μm; PM_2.5_: Mass concentration of particles less than 2.5 μm; PNC: Particle number concentrations; RUPIOH: Relationship between Ultrafine and fine Particulate matter in Indoor and Outdoor air and respiratory Health; SOPs: Standard operating procedures; 95% CI: 95% confidence interval.

## Competing interests

The authors declare that they have no competing interests.

## Authors’ contributions

All authors of this paper have critically read and approved the final version submitted. They have also made substantive intellectual contributions by directly participating either in the planning, execution, or analysis of the study. AK contributed to the development of the study design, acquisition and interpretation of data and drafted the paper. AA did the analysis, contributed to the interpretation of data and wrote the statistical analysis section of the paper. DP, IGK, JJdeH contributed substantially to acquisition and interpretation of data. JGA, RMH, AK, JP, KH, GPAK, KK contributed to the study design, interpretation of data and have been involved in drafting the manuscript. GH conceived and developed the study design, contributed to the interpretation of data and was involved in drafting the paper. All authors have revised drafts and contributed to the revisions.
